# Enhancing walking efficiency of adolescents with neurological impairments using an exosuit for ambulatory activities of daily living

**DOI:** 10.3389/frobt.2024.1335733

**Published:** 2024-03-13

**Authors:** Chiara Basla, Giulia Mariani, Peter Wolf, Robert Riener, Hubertus J. A. van Hedel

**Affiliations:** ^1^ Sensory-Motor Systems (SMS) Lab, Institute of Robotics and Intelligent Systems (IRIS), ETH Zürich, Zürich, Switzerland; ^2^ Swiss Children’s Rehab, University Children’s Hospital Zurich, University of Zurich, Affoltern am Albis, Switzerland; ^3^ Children’s Research Center, University Children’s Hospital Zurich, University of Zurich, Zurich, Switzerland; ^4^ Spinal Cord Injury Center, Balgrist University Hospital, Medical Faculty, University of Zürich, Zurich, Switzerland

**Keywords:** youths, assistive device, outdoor walking, participation, user’s perspective

## Abstract

**Introduction:** Children and adolescents with neurological impairments face reduced participation and independence in daily life activities due to walking difficulties. Existing assistive devices often offer insufficient support, potentially leading to wheelchair dependence and limiting physical activity and daily life engagement. Mobile wearable robots, such as exoskeletons and exosuits, have shown promise in supporting adults during activities of daily living but are underexplored for children.

**Methods:** We conducted a cross-sectional study to examine the potential of a cable-driven exosuit, the Myosuit, to enhance walking efficiency in adolescents with diverse ambulatory impairments. Each participant walked a course including up-hill, down-hill, level ground walking, and stairs ascending and descending, with and without the exosuit’s assistance. We monitored the time and step count to complete the course and the average heart rate and muscle activity. Additionally, we assessed the adolescents’ perspective on the exosuit’s utility using a visual analog scale.

**Results:** Six adolescents completed the study. Although not statistically significant, five participants completed the course with the exosuit’s assistance in reduced time (time reduction range: [-3.87, 17.42]%, *p*-value: 0.08, effect size: 0.88). The number of steps taken decreased significantly with the Myosuit’s assistance (steps reduction range: [1.07, 15.71]%, *p*-value: 0.04, effect size: 0.90). Heart rate and muscle activity did not differ between Myosuit-assisted and unassisted conditions (*p*-value: 0.96 and 0.35, effect size: 0.02 and 0.42, respectively). Participants generally perceived reduced effort and increased safety with the Myosuit’s assistance, especially during tasks involving concentric contractions (e.g., walking uphill). Three participants expressed a willingness to use the Myosuit in daily life, while the others found it heavy or too conspicuous.

**Discussion:** Increased walking speed without increasing physical effort when performing activities of daily living could lead to higher levels of participation and increased functional independence. Despite perceiving the benefits introduced by the exosuit’s assistance, adolescents reported the need for further modification of the device design before using it extensively at home and in the community.

## 1 Introduction

Participation and functional independence are pivotal in enhancing quality of life and fostering holistic development, enabling people to engage in activities actively (Jain et al., 2007). Involvement in daily life and self-sufficiency are often limited for children and adolescents with walking impairments (Claassen et al., 2011). It was previously shown that children with a disability affecting their lower limbs are less physically active than their aged-matched healthy peers, performing fewer steps in everyday life (Bjornson et al., 2007). Increased effort in walking is the major contributor to reduced physical activity (Fowler et al., 2007). Many childhood-onset disorders, such as cerebral palsy (CP), traumatic brain injury (TBI), and stroke, cause abnormalities in the walking pattern, forcing children to walk slower, with increased inter-limb asymmetry, reduced stability, earlier onset of fatigue, and higher metabolic cost compared to typically developing peers (García et al., 2016). In a previous study run at the Swiss Children’s Rehab (University Children’s Hospital Zurich, Affoltern am Albis, Switzerland) (Rast and Labruyère, 2020), it has been shown that these pathologies represent more than 50% of the clinic population. Furthermore, according to the Disability Report from UNICEF, children with moderate to severe walking difficulties account for approximately 2.5% of the children between the ages of 5 and 17 (United Nations, 2021).

Given the high prevalence of moderate to severe lower limb impairments, improving or recovering walking ability is a frequent goal in pediatric neurorehabilitation (Rast and Labruyère, 2020). However, performing physical activity cannot be limited to in-clinic rehabilitation; it is fundamental to maintain an active lifestyle within the domestic and community environment to counteract the gradual decline in physical condition over time and ensure adequate participation in activities of daily living (ADLs).

At present, to stay mobile across ADLs, assistive devices, such as crutches and rollators, are pivotal for individuals with walking impairments. Still, these assistive devices offer limited support, which may not adequately meet the needs of children with moderate to severe disabilities. As a result, these children might need a wheelchair for their daily mobility, particularly for longer distances, limiting their ability to engage in physical activities and participate in everyday life.

To resolve this concern, mobile, wearable robots providing active support in daily life, such as exoskeletons and exosuits, have been recently developed for patients with walking impairments. Although they have been intensively investigated for the adult population (Asbeck et al., 2015; Awad et al., 2017; Kim et al., 2019; Awad et al., 2020; Basla et al., 2022), few mobile wearable robots have been developed for and evaluated in the pediatric population. The Hybrid Assistive Limb (HAL) exoskeleton was downsized and tested with approximately 20 children with CP showing improvements in self-selected walking speed, cadence, and hip joint angles (Matsuda et al., 2018; Mataki et al., 2020). Lerner and colleagues developed a knee exoskeleton for children with CP and demonstrated improved walking patterns and reduced muscle activity in seven children (Lerner et al., 2017a; Lerner et al., 2017b; Lerner et al., 2017c). The same research group also developed a lightweight cable-driven exosuit to assist ankle plantar and dorsiflexion while walking in children with CP (Lerner et al., 2018). Tests with 15 participants demonstrated reduced metabolic cost during walking, increased walking speed, and reduced soleus muscle activity (Lerner et al., 2018; Orekhov et al., 2020). The ATLAS exoskeleton was developed to support small kids with severe spinal muscular atrophy (SMA) (Cumplido-Trasmonte et al., 2022). It showed improved hip flexors and extensors strength and increased hip range of motion in three participants (Cumplido-Trasmonte et al., 2022). A single-case study including a 6-year-old boy with SMA evaluated the feasibility of the ATLAS to train walking at home (Garces et al., 2022). The ATLAS and the HAL exoskeletons provide mechanical support to all three leg joints (hip, knee, and ankle) and weight 12 kg and 14 kg, respectively. Lighter devices (between 2 kg and 6 kg), such as the knee and the ankle exoskeletons developed by Lerner and colleagues, provide support to single joints. Lightweight exoskeletons that provide assistance to multiple joints (e.g., hip and knee) have not been tested in the pediatric population yet, despite it was previously shown that assisting multiple joints leads to increased metabolic benefits compared to single joints exoskeletons (Franks et al., 2021). Additionally, due to the excessive weight and bulkiness or the early prototyping phase, all the technologies mentioned above were mainly tested within laboratory settings, either over a treadmill or along short level ground distances. During the home study with ATLAS, all training sessions consisted of level ground walking, both due to the device’s design and the severe walking impairment of the participant. Therefore, it remains unclear if children with neurological gait impairments can use these exoskeletons for more complex ambulatory tasks of daily living (such as climbing stairs or walking uphill). Finally, these studies never investigated what the children’s perspective is about using such devices at home and in the community. Previous studies have assessed the viewpoints of clinicians and parents regarding the application of robotic exoskeletons outside the clinical setting (Borenstein et al., 2020), but the subjective experiences of the children remained unexplored.

The Myosuit (Myoswiss AG, Switzerland) is a lightweight exosuit that provides mechanical support to the hip and the knee joints simultaneously. Its practicality for in-home and community-based training and engagement in ADLs was previously shown for the adult population (Basla et al., 2022). Leveraging this knowledge, we designed the current study to assess the effectiveness of biarticular mechanical support provided by the exosuit while walking in- and outdoors in children with diverse ambulatory limitations. Primarily, the study evaluated the effectiveness of the exosuit’s mechanical support in improving walking efficiency. Secondly, we explored the benefits perceived by adolescents and their attitude towards adopting the exosuit at home and in the community. We focused on adolescents as the exosuit was originally developed for the adult population. This cross-sectional study included three familiarization sessions and a final assessment during which the participants repeatedly walked a course that included various walking tasks with and without the support of the exosuit. In this study, we evaluated the changes induced by the assistance of the exosuit, rather than the clinical utility of the device, getting insights on the benefits of biarticular mechanical support in ambulatory activities of daily living. The findings will guide the development of a new version of the exosuit specifically designed for the pediatric population.

## 2 Methods and materials

### 2.1 Wearable robot

**FIGURE 1:**
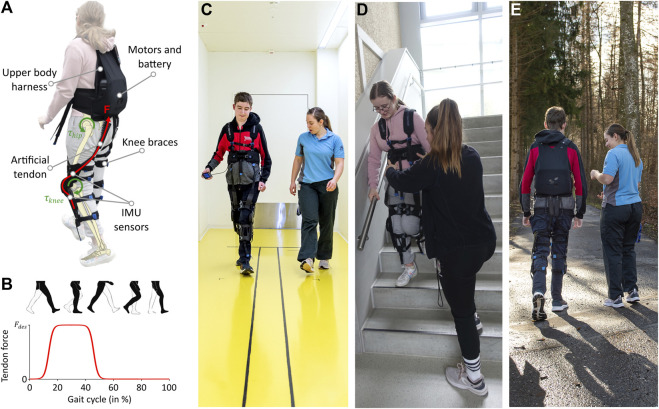
The Myosuit. **(A)** Main components of the Myosuit, which is a lightweight exosuit that assists walking. **(B)** The motors provide the desired force (F_des_) during the stance phase (10%–50%) of gait by tensioning the cables routed along the legs and extending the hip and the knee joints. **(C–E)** Participants walking in various environments with the Myosuit during the familiarization sessions.

The study utilized the Myosuit Gamma (Myoswiss AG, Switzerland), a cable-driven exosuit that assists people with lower limb impairment to walk and climb stairs. It consists of two knee orthoses and a backpack containing the electronics, two motors, and a battery (Figure 1A). One cable is routed from each motor, over the buttock, around the thigh, and fixed at the shank. The motor, when activated, retracts the cable and extends the hip and knee joints simultaneously. Forces are applied during the stance phase of the gait when the leg is bearing the body weight. Inertial measurement units (IMU) are used to detect the gait events (heel strike and toe off) (Figure 1B). The desired force can be set up through a user controller and adjusted over six discrete levels that correspond to a portion (0, 20, 40, 60, 80, and 100%) of the maximally available assistance (230 N (Haufe et al., 2020a)). The Myosuit was initially designed and CE-marked for the adult population and can be used by people between 150 and 195 cm tall and weighing 45–110 kg. Therefore, we only included in the study adolescents who fulfilled these criteria.

### 2.2 Participants

We established the following inclusion criteria: 1) age between 10 and 18 years old, 2) height between 150 and 195 cm and weight between 45 and 110 kg (as per the Myosuit’s Instruction of Use manual), 3) confirmed diagnosis of a neurological pathology that leads to gait impairment, 4) ability to communicate discomfort or pain, 5) ability to walk 10 m without the assistance of a person (traditional assistive devices were allowed, if necessary), 6) understand and answer verbal questions. We excluded adolescents with skin irritations underneath the exosuit’s cuffs. We submitted the study protocol to the Ethical Committee of the Canton of Zurich (BASEC-Nr. Req-2023–00038), which confirmed that the study did not fall within the scope of the Human Research Act. According to Good Clinical Practice guidelines, we informed the participants and their legal guardians verbally and in writing about the study’s content and procedure. Each participant and the legal guardian provided written informed consent before participating in the study.

### 2.3 Study design

We conducted this cross-sectional study at the Swiss Children’s Rehab. Participants took part in multiple sessions. In the first evaluation session, they were characterized by age, gender, diagnosis, severity, body weight and body height and regularly used walking aids and orthoses. Participants also performed a 10-m walk test (10MWT) at their maximal speed, from which we computed their baseline walking speed. Furthermore, we performed the Gillette Functional Assessment Questionnaire (GFAQ) to evaluate the participant’s walking ability (Novacheck et al., 2000). The GFAQ assesses walking abilities on a scale from 1 (“The child cannot take any steps at all”) to 10 (“The child walks, runs, climbs on level and uneven terrain without difficulty or assistance”). Subsequently, a trained therapist fitted the device to the child and adjusted it to the participant’s body size. Finally, the adolescents tried to walk with the device’s assistance, and the therapist evaluated whether using the device was safe and if the study could be continued. The therapist would interrupt the procedures if the participant, the therapist, or another person were in danger.

If the therapist considered the application of the device safe, the participant took part in three 45-min familiarization sessions spread over one or 2 weeks. Each familiarization session was structured to include different ambulatory tasks in line with the individual goal of each participant (Figures 1C–E). We considered the familiarization with the device fundamental for the participants to habituate themselves to the assistance of the technology and for obtaining reliable quantitative results (Haufe et al., 2021).

**FIGURE 2:**
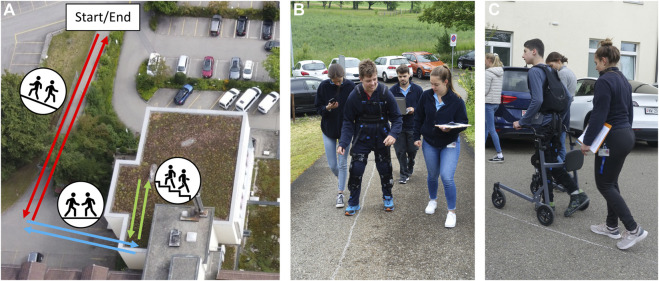
The course. **(A)** The course in the backyard of the Swiss Children’s Rehab (University Children’s Hospital Zurich, Affoltern am Albis, Switzerland). It comprises six subtasks: uphill, level ground, upstairs, downstairs, level ground and downhill walking. **(B, C)** Participants walking different portions of the course (uphill and level ground, respectively) during one of the trials of the final assessment.

Finally, in the assessment session (Figure 2), each adolescent walked an approximately 160 m long course in the backyard of the Swiss Children’s Rehab. The course included various subtasks that mimic scenarios one might experience during community walking: walking up- and downhill, walking level ground, and climbing stairs up and down (Figure 2A). Each participant repeated the course four times (referred to as trials), twice with and twice without the assistance of the Myosuit. The participants wore the Myosuit in all trials. This choice allowed us to remove weight as a confounding factor and focus on the benefits of the assistance itself; in fact, since the Myosuit was initially developed for the adult population, we feared that its weight could have confounded the benefits of the device’s mechanical support when used with adolescents (whose weight was close to the lower allowed limit of 45 kg). Therefore, the exosuit was on (Myo ON) or off (Myo OFF), respectively. The two conditions (Myo ON and Myo OFF) were performed in an ABBA order to compensate for the effect of fatigue, and the assignment of conditions to A and B was randomized between participants. We instructed the participants to “walk as fast but safe as possible without running” and “take breaks whenever needed”. To guarantee comparability between the two conditions, the patients used the same assistive aids in all trials. Between each trial, each participant took a break (approx. 5 min) to avoid carried-over fatigue.

To assess the participants’ perspective on the Myosuit’s utility, the participants walked the course once during the final familiarization session. During this trial, the Myosuit assisted half of each subtask, while in the other half the assistance was turned off. On/off sequence was again randomized. The participants were verbally made aware of the change in the Myosuit’s assistance. After each subtask, we asked the participants to rate on an 11-point visual analog scale (VAS) the difference in fatigue and safety when the support of the Myosuit was provided (−5 being “much more strenuous” and “much more unsafe”, 0 being “same”, and +5 being “much less strenuous” and “much safer”). Finally, participants rated whether they would use the Myosuit in their everyday life from 0 (“never”) to 10 (“always”) and argued in which everyday life situation they would use it.

### 2.4 Outcome measures and data processing

We recorded the time needed to complete the course with a stopwatch and counted the number of steps during uphill, level ground, and downhill walking. In a Case Report Form, we noted the number and duration of breaks. To estimate the effort while walking in the different conditions, we measured the heart rate (HR) during the whole course with the Polar H10 chest band connected to a mobile phone running the Polar Beat App (Polar Electro Oy, Finland). For the data analysis, we computed the average HR per trial over the entire duration of the course. Additionally, we recorded the electromyographic (EMG) activity of the vastus lateralis, semitendinosus, and gastrocnemius medialis, for both legs. These muscles were selected to gain a better understanding of the entire walking biomechanics. In the analysis, we primarily focused on the vasti lateralis since they work in parallel with the assistance provided by the Myosuit (i.e., extending the knee joint). We used self-adhesive foam and hydrogel Kendall electrodes (30 × 24 mm, Covidien, Mansfield, USA). We prepared the skin and applied the EMG according to the SENIAM guidelines (Hermens et al., 2000). However, the position of the electrodes might have varied slightly from the guidelines due to the cuffs and straps of the Myosuit. We recorded the signal at 1,500 Hz with the wireless TeleMyo DTS Telemetry System and the MyoResearch XP software (Noraxon, Scottsdale, USA). We processed the EMG data in Python (version 3.8.10). We rectified the raw EMG data and used a moving average filter with a time window of 100 ms. Subsequently, we computed the average EMG amplitude per muscle and trial. We normalized it by the average muscle activity during level walking without the Myosuit’s assistance to ensure comparability between participants. Finally, we averaged the normalized muscle activity of the corresponding muscles of the two legs.

### 2.5 Statistical analysis

We used the fastest trial per condition in the analysis. All statistical computations and analyses were conducted in R (version 4.3.1) using RStudio IDE (RStudio Team, 2023. RStudio, Inc., Boston, USA). We used the Shapiro-Wilk test to evaluate the data distributions, i.e., the differences between the two conditions of the time needed to complete the course, the number of steps, the average HR, and the normalized EMG amplitude. We applied the paired t-test to evaluate the significance of the difference between the two conditions if the data were normally distributed. We applied the Wilcoxon signed-rank test if the data were not normally distributed. Statistical significance was determined using an alpha threshold of 0.05.

Additionally, we computed the effect size to estimate the magnitude of the differences found. We determined the Cohen’s d for normally distributed data by dividing the mean of the differences between conditions by their standard deviation. If data were not normally distributed, we determined the Wilcoxon effect size r by dividing the z statistic of the Wilcoxon signed-rank test by the square root of the sample size. According to previously published literature (Cohen, 1988; Tomczak and Tomczak, 2014), the effect size was interpreted as follows: small effect for 0.2≤d< 0.5 and 0.1≤r< 0.3 , medium effect for 0.5≤d< 0.8 and 0.3≤r< 0.5 , and large effect for d≥0.8 and r≥0.5 .

## 3 Results

### 3.1 Participants

Seven adolescents fulfilled the inclusion criteria and participated in the study (demographic data provided in Table 1). Three participants were diagnosed with TBI, each with various walking abilities and cognitive functioning. Three participants were diagnosed with CP, with Gross Motor Function Classification System (GMFCS) levels I to III. One participant was diagnosed with hereditary spastic paraparesis (HSP). The participants spanned a broad spectrum of walking ability levels, from 3 to 9 in the GFAQ. Six out of seven participants were undergoing inpatient rehabilitation at the Swiss Children’s Rehab. Among the participants, P4 and P6 exhibited mild cognitive impairments. Participant P4 did not complete the entire study protocol due to feeling unsafe with the device and was excluded from the analysis.

**TABLE 1:** Participants’ demographics.

ID	Age (years)	Sex	Height (cm)	Weight (kg)	Diagnosis	10MWT (sec)	GFAQ	Aids/Assistance	Cognition
P1	12.6	F	158	64	TBI	9.0	8	Close supervision	Inconspicuous
P2	14.7	M	176	54	CP (III)	10.5	4	Posterior walker	Inconspicuous
P3	14.4	M	168	47	TBI	6.7	9	Close supervision	Inconspicuous
P4	15.9	F	175	55	TBI	34.9	3	Taurus walker	Lightly impaired
P5	17.7	F	164	63	HSP	9.3	9	Forearm crutches	Inconspicuous
P6	14.8	M	174.5	83	CP (II)	17.9	9	Close supervision	Lightly impaired
P7	14.7	M	173.5	49.5	CP (I)	8.08	7	Close supervision	Inconspicuous

F, female; M, male; TBI, traumatic brain injury; CP, cerebral palsy (in brackets, the GMFCS, level); HSP, hereditary spastic paraparesis; 10MWT, 10 m walk test; GFAQ, gillette functional assessment questionnaire.

### 3.2 Time and number of steps to complete the course

**FIGURE 3:**
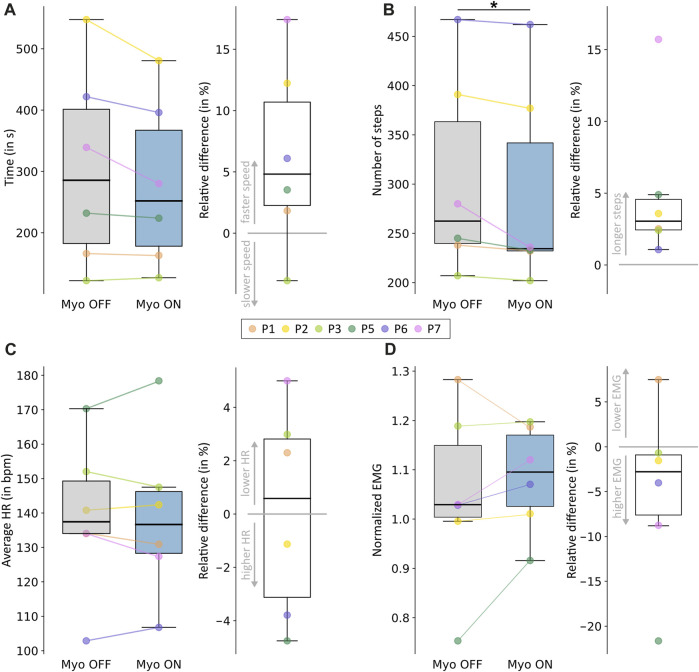
Outcome measures to evaluate walking efficiency. **(A)** The time needed to complete the course. **(B)** The number of steps required to complete the course. **(C)** Average heart rate over the entire course. **(D)** Normalized muscle activity of the vastus lateralis over the whole course. Each panel shows the results for the Myo OFF and Myo ON conditions on the left and the percentage differences between the two conditions on the right. Positive differences correspond to a decrease in the outcome, considered an improvement in walking efficiency. Negative differences correspond to an increase in the outcome, considered a worsening in walking efficiency. Individual responses for each participant are reported using different colors. Statistically significant differences are shown in the figure.

The time needed to complete the course decreased for all but one participant when the Myosuit’s assistance was on compared to off (Figure 3A; Supplementary Table S1). On average, participants required 304.9 s (standard deviation (SD) 162.19 s) in the off condition and 278.4 s (SD 137.1 s) in the on condition. The percentage time decrease during Myo ON compared to Myo OFF varied between −4% and 18%. The t-test results showed no significant difference between the two conditions (*p* = 0.08), but the effect size was large (d = 0.88).

The number of steps taken during the course decreased for all participants when the Myosuit’s assistance was on compared to off (Figure 3B; Supplementary Table S1). On average, participants took 304.7 steps (SD 102.0 steps) in the off condition and 290.3 steps (SD 104.3 steps) in the on condition. The percentage decrease in number of steps during Myo ON compared to Myo OFF varied between 1% and 16%. The Wilcoxon’s signed-rank test results showed a significant difference between the two conditions (*p* = 0.04), and the effect size was large (r = 0.90).

### 3.3 Heart rate and EMG activity

The mean HR was 138.9 beats per minute (bpm) (SD 24.0 bpm) with the Myosuit’s assistance and 139.02 bpm (SD 22.4 bpm) without assistance (Figure 3C; Supplementary Table S2). Out of the six participants, three showed an increase in average HR when walking with the assistance of the Myosuit, and three showed a decrease. The percentage decrease in average HR during Myo ON compared to Myo OFF varied between −5% and 5%. The paired t-test results showed that the difference between the two conditions was not significant (*p* = 0.96), and the effect size was small (d = 0.02).

The mean normalized muscle activity of the vastus lateralis was 1.00 (SD 0.16) with the Myosuit’s assistance and 1.03 (SD 0.09) without assistance (Figure 3D; Supplementary Table S2). Out of the six participants, five showed an increase in muscle activity when walking with the assistance of the Myosuit. The percentage decrease in average muscle activity during Myo ON compared to Myo OFF varied between −22% and 7%. The paired t-test results showed that the difference between the two conditions was not significant (*p* = 0.35), and the effect size was small (d = 0.42). We obtained similar results for the semitendinosus and the gastrocnemius and reported them in the Supplementary Material S1.

### 3.4 Adolescents’ perception during various ambulatory tasks

**FIGURE 4:**
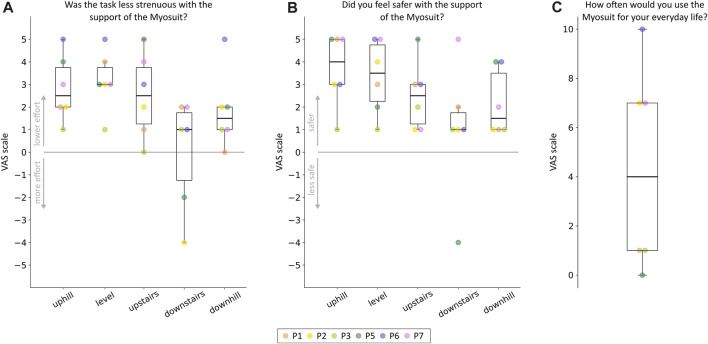
Adolescents’ perception of the exosuit’s assistance. Participants’ ratings on the perceived usefulness of the device in terms of **(A)** effort reduction and **(B)** safety increase. The ratings are reported for each subtask of the course separately. **(C)** Participant’s ratings on the willingness to use the device in everyday life. Individual responses for each participant are reported using different colors.

On average, participants reported reduced perceived effort and increased safety when the Myosuit’s assistance was provided (Figures 4A, B). The Myosuit’s assistance was better perceived in subtasks that required higher concentric contractions (namely, uphill and level walking and upstairs climbing) than those requiring eccentric contractions (namely, downhill walking and downstairs climbing). Participant P2 reported the lowest perceived benefit in effort and safety. Participants P6 and P7 reported, on average, the highest scores in both domains. Three participants (P2, P6, and P7) said they would extensively use the Myosuit in everyday life (Figure 4C). They mentioned walking at home, uphill, and outdoors for longer distances as activities they would perform with the exosuit. The other three participants showed no interest in using the exosuit outside the clinical environment. Particularly, participant P5 mentioned that the exosuit was too conspicuous to be used in daily routines. Participant P2 also brought up this limitation. In addition, multiple participants reported weight and donning as limiting factors.

## 4 Discussion

### 4.1 A trend towards increased walking efficiency

The study demonstrated that the Myosuit’s assistance resulted in faster walking speeds in five of six participants. Although the increase in walking speed induced by the Myosuit’s assistance was not statistically significant, the large effect size suggests that the exosuit can enhance speed and efficiency during various walking tasks. This improvement could be attributed to increased strength in the legs, stability, or safety (Hoffman et al., 2018). These results are consistent with previous research by Haufe and colleagues, which also showed the Myosuit’s ability to increase the walking speed of an adult participant with a spinal cord injury (Haufe et al., 2020b). On the other hand, the P. REX knee exoskeleton showed a slight decrease in walking speed with one 15-year-old boy with CP compared to zero torque condition (Chen et al., 2021). However, the single-case nature of the study makes comparisons difficult. Given that children with neurological impairments typically exhibit slower walking speeds than their healthy peers (García et al., 2016), enabling faster walking might be a valuable incentive to boost engagement in the community. Since motor function was a predictor for participation restrictions in children with CP (Beckung and Hagberg, 2007), faster walking speeds could lead to higher levels of participation and increased functional independence (Schenker et al., 2005; Moreau et al., 2016; Faria-Fortini et al., 2019).

The assistance of the Myosuit allowed participants to complete the course in reduced time by enabling longer steps. This result is promising as children with neurological disorders tend to walk with reduced step length compared to age-matched healthy controls (Abel and Damiano, 1996). This improvement in step length may be attributed to improved balance and increased feeling of safety provided by the Myosuit’s assistance (as rated on the VAS by each participant). Additionally, the Myosuit’s support to the hip and knee extensor muscles might have been crucial for enhancing walking mechanics since these muscles largely contribute to increased step length (Lim et al., 2017). Our findings diverged from the results of studies with a tethered knee exoskeleton (Lerner et al., 2017a; Lerner et al., 2017c) and the P. REX exoskeleton (Chen et al., 2021), which reported no significant differences in step length compared to assistance off. Compared to a cable-drive exosuit (such as the Myosuit), these rigid exoskeletons have higher distal masses, which might have hindered the participant from taking longer steps. Longer steps are associated with a more efficient gait pattern (Rose et al., 1994; Kimoto et al., 2017) and could enhance functional independence for children with walking impairments.

Usually, an increase in walking speed is accompanied by an increase in HR (Waters et al., 1988), which can be interpreted as an increase in energy expenditure (Green, 2011). In our study, we observed no changes in the average HR (i.e., metabolic demand) despite the increased walking speed, suggesting that the assistance provided by the Myosuit contributed to a more efficient muscle activation pattern. Reducing fatigue and increasing endurance are fundamental factors for an assistive device and might translate to greater independence and increased participation for children with walking impairments (Orekhov et al., 2020; Hunt et al., 2022).

The average muscle activity of the vastus lateralis was not affected by the Myosuit’s assistance. Similarly to our findings, Lerner and colleagues did not find any significant difference in muscle activity of the vastus lateralis when comparing the assistance of a tethered knee exoskeleton to assistance off (Lerner et al., 2017c). Our results for muscle activity are very individual, and it is challenging to elaborate a comprehensive conclusion. Furthermore, many factors might have influenced the EMG results, primarily the different walking speeds in the two conditions. Walking speed highly affects EMG activity (Hof et al., 2002) and might have confounded any decrease in muscle activity due to the Myosuit’s assistance. All previous studies evaluating changes in muscle activity due to the assistance of a robotic device were run in very controlled environments, usually walking on a treadmill at the same controlled speeds (Panizzolo et al., 2016; Lerner et al., 2017c; Chen et al., 2021; Franks et al., 2021).

### 4.2 Divergent perspectives on the usability of the Myosuit at home and in the community

The willingness to use the device in daily life related to the improvements during the course. Participants P2, P6, and P7 showed the most remarkable advances in walking speed and reported they would extensively use the Myosuit in everyday life. It was also related to the perceived reduction in effort and increased safety for participants P6 and P7. Participant P2 perceived the support as helpful during uphill and level walking but felt more restricted in upstairs climbing and eccentric tasks, probably due to the participant’s lower residual functional abilities and greater difficulties in performing the tasks. The activities that participants mentioned they would perform with the Myosuit aligned with the individual goal of each participant. Participant P2 had low residual functional abilities and mainly relied on a wheelchair for daily mobility. He would use the Myosuit to be more physically active inside the house. Participants P6 and P7 navigate freely at home and in the community and would use the Myosuit to increase endurance outdoors. The other participants benefited minimally from the Myosuit during the course and did not foresee taking the Myosuit home. Participants mentioned many factors that need to be improved before extensive use. As expected, they perceived weight as the main limiting factor. Participants also mentioned the need for a less conspicuous device and easier donning. Device appearance played a fundamental role for participant P5. Although she could perceive the benefits of the Myosuit’s support, she would never use the device in public. Interestingly, in a previously conducted study, adult users of the Myosuit mentioned only the difficulty in donning and did not perceive the weight and appearance as obstacles to a higher rate of adoption (Basla et al., 2022). These findings highlight the differences in requirements to be considered when developing technologies for the pediatric population.

### 4.3 Variability in patients’ reactions to the exosuit’s assistance

In this exploratory study, we included a heterogeneous population regarding diagnoses and walking ability, which resulted in variable responses to the exosuit’s assistance.

Haufe and colleagues recommended the utilization of the Myosuit with adults who regularly employ walking aids or exhibit baseline walking speeds lower than 1 m/s (Haufe et al., 2020a). These recommendations also seem to hold for adolescents. Participant P2, who walked at 0.95 m/s with a posterior walker, showed one of the greatest increases in walking speed, i.e., more than 10%. Similarly, participants P6 (baseline walking speed 0.56 m/s) and P5 (walking with forearm crutches) also required less time to complete the course. Participant P3 was already fast at baseline (1.49 m/s), did not need any assistive aid, and was slightly hindered by the assistance of the exosuit. Due to the limited support the exosuit provides, patients with little residual walking abilities might not be able to use or benefit from it. This was the case for participant P4, whose considerably reduced walking speed and the need to concentrate highly on every step prevented her from accustoming to the Myosuit’s assistance. Despite fast baseline walking without assistive aid, participant P7 showed the greatest improvement due to the assistance of the exosuit. Participant P7 suffered unilateral CP that resulted in muscle weakness in one leg. At the moment of the study, he was recovering from pain-alleviating surgery of the foot of the more affected leg. The Myosuit showed promise in supporting patients who cannot walk safely due to problems in loading one leg.

Relying solely on the baseline 10MWT may not be adequate for identifying children who would benefit more from an exosuit. We found no correlation between baseline 10MWT and the improvement due to the exosuit’s assistance. On the contrary, the participants who took longer to complete the course without the assistance of the Myosuit showed greater improvements. Interestingly, the three participants who showed the most substantial improvements were diagnosed with CP. This observation is consistent with the predominant focus of exoskeleton research on children with CP. Most therapeutic and assistive exoskeletons for children were developed for and tested with individuals with CP experiencing crouch gait. In our study, we showed that the Myosuit could support patients exhibiting a wide variety of symptoms, such as reduced muscular strength and reduced stability when walking.

### 4.4 Need for a pediatric version

Despite the trend towards improved walking efficiency, the absolute improvements in time and number of steps were moderate and presumably not clinically meaningful. The device design might have constrained the potential for more favorable results. Indeed, the Myosuit was not designed for children and adolescents, which limited the number of participants fulfilling the inclusion criteria. These considerations point toward the need for a new version of the exosuit explicitly designed for the pediatric population. Our future work will focus on making the technology available to a larger cohort of children. The necessity for a more versatile design stems from several compelling reasons that emphasize the importance of tailoring the exosuit to suit the individual needs of young users. In this direction, we will develop an exosuit that accommodates a broader range of body sizes, considering that children with neurological disorders often exhibit stunted growth compared to healthy peers (Stevenson et al., 2006). The device weight inevitably represented a limitation for the study and should be reduced to less than 10% of the body mass of the intended target group (Mackenzie et al., 2003).

Furthermore, applying an exosuit not designed for the tested population resulted in an overpowered design and precluded the possibility of precisely tuning the assistance to the participants’ needs. The need for reduced motors’ power will help us accomplish the weight requirements and guarantee finer tuning of the support levels. Additionally, the exosuit should be able to accurately detect extremely short and irregular steps and precisely time their actuation. Children, more than adults, might be hindered by wrongly timed assistance, not only in performing the movement but especially in accepting the device. Finally, hyperextension is a common gait abnormality in children with neurological conditions. Hyperextension is often a compensatory mechanism to achieve stability or maintain balance during walking. An exosuit with the same working principle as the Myosuit (i.e., hip and knee extension during the stance phase of gait) could provide the stability and confidence that these children are lacking while loading the leg and help them walk with a more physiological gait pattern Avoiding hyperextension prevent chronic joint problems over time and injuries. Despite the incorporated mechanical end-stop that prevents the orthosis from bending over 180°, the compliance of the Velcro fasteners still allows participants to hyperextend their knees. A design with increased stiffness would assist in getting rid of this abnormality of their walking pattern. Despite the apparent benefits of not hyperextending while walking, previous studies have not investigated the possibility of relearning a more physiological walking pattern through the assistance of an exosuit.

### 4.5 Study limitations

We acknowledge the small sample size, which was influenced by strict inclusion criteria on the body size of the participants. However, the sample size in this study is consistent with the number of participants included in previously published studies that evaluated rigid exoskeletons on the same target group (Lerner et al., 2017b; Lerner et al., 2017c; Lerner et al., 2018; Orekhov et al., 2020; Sanz-Merodio et al., 2020; Delgado et al., 2021; Cumplido-Trasmonte et al., 2022). We intentionally included a heterogeneous sample due to the study’s exploratory nature. This choice may have on the one hand influenced the overall outcomes and masked potential trends in the results, but, on the other hand, was extremely relevant to give the therapists and researchers an idea of the target group that benefitted the most from the technology. Moreover, the study inclusion criteria, forced by device design constraints, not only restricted the recruitment to adolescents but also resulted in the exclusion of all adolescents with crouch gait having inpatient rehabilitation at the moment of the study due to their limited weight. Due to its working principle, the Myosuit should be advantageous for patients facing weaknesses in anti-gravity muscles (Schmidt et al., 2017). Further testing is needed to evaluate the benefits of the Myosuit’s support in a more homogeneous group of patients with crouch gait.

Furthermore, as explained in the method section, we did not consider the condition in which the participants did not wear the device. An additional third condition (i.e., without wearing the device) would have been too exhausting for the participants. A previous study has shown that the Myo OFF condition did not influence walking kinematics in healthy adults walking on a treadmill compared to the no-suit condition (Haufe et al., 2019). However, although the device does not seem to hinder the biomechanics of walking when no assistance is provided, we would need to confirm these results in pediatric patients in more ecological settings. We especially feared that the weight of the current device would have influenced the participants’ performance and confounded the improvements due to the provided support. The feedback from the participants confirmed our concern about the device’s weight. A study that compares the Myosuit to the condition of not wearing the device will be performed after the pediatric version has been developed.

Finally, performing the study outside a laboratory setting allowed us to test endurance in common everyday life activities and provide children with a more authentic representation of the challenges they face in daily life. However, it's important to note that, despite the study was performed outdoors, its methodology maintained a high level of structure, resembling a controlled setting in terms of repeatability and absence of irregularities in the protocol. This structured setup, while beneficial for controlled experimentation, limited our ability to draw conclusions about the long-term usability of the exoskeleton in home and community settings. To address this limitation, further research is required using the exoskeleton in a domestic environment with a less rigid protocol.

## 5 Conclusion

This study highlighted the potential benefits of the Myosuit in supporting adolescents’ mobility during indoor and outdoor walking. The study showed large effects towards increased walking speed and larger steps without changes in walking effort. However, it demonstrated high variability among participants concerning walking efficiency improvements. The findings underscored the importance of developing a dedicated pediatric version of the Myosuit, which should address weight constraints and allow fine-tuned assistance. The variability in participants’ responses emphasized the importance of tailoring technology to suit children’s needs. Overall, this study represented an initial step toward empowering youths with walking impairments to lead more active and engaged lives.

## Data Availability

The raw data supporting the conclusion of this article will be made available by the authors, without undue reservation.

## Abbreviations

10MWT, 10 m Walk Test; ADL, Activity of Daily Living; bpm, beats per minute; CP, Cerebral Palsy; EMG, Electromyography; GFAQ, Gillette Functional Assessment Questionnaire; GMFCS, Gross Motor Function Classification System; HAL, Hybrid Assistive Limb; HR, Heart Rate; HSP, Hereditary Spastic Paraparesis; IMU, Inertia Measurement Unit; SMA, Spinal Muscular Atrophy; TBI, Traumatic brain injury; VAS, Visual Analogue Scale.
