# Supplementary material for: Enhancing walking efficiency of adolescents with neurological impairments using an exosuit for ambulatory activities of daily living
Source: Front Robot AI. 2024 Mar 13;11:1335733. doi: 10.3389/frobt.2024.1335733 (PMC10976852; doi:10.3389/frobt.2024.1335733)
Supplement: Supplementary file 1 [file DataSheet1.pdf]

Supplementary Material

Enhancing walking efficiency of adolescents with neurological impairments using an exosuit for ambulatory activities of daily living

Chiara Basla[1,2,3*], Giulia Mariani[1,2,3], Peter Wolf[1], Robert Riener[1,4], Hubertus van Hedel[2,3]

*** Correspondence:** Chiara Basla chiara.basla@hest.ethz.ch

Table S1 shows the time and number of steps taken to complete the course per participant and condition. Table S2 shows the average heart rate and normalized muscle activity of the vastus lateralis per participant and condition. Table S3 shows the average normalized muscle activity of the semitendinosus and the gastrocnemius per participant and condition. EMG of the semitendinosus was discarded for participants P3 and P7 and of the gastrocnemius for participant P7 due to noise in the signals. The results of Table S3 are graphically represented in Figure S1. The paired t-test results on the semitendinosus data showed no significant difference between the two conditions ($p = 0.94$), and the effect size was small ($d = 0.04$). The paired t-test results on the gastrocnemius data showed no significant difference between the two conditions ($p = 0.18$), and the effect size was medium ($d = 0.71$). Absolute and percentage decreases during the Myo ON condition compared to the Myo OFF condition were computed. Positive differences correspond to a decrease in the outcome, considered an improvement in walking efficiency. Negative differences correspond to an increase in the outcome, considered a worsening in walking efficiency.

1 Supplementary Tables

Table S1. Time and number of steps to complete the course.

ID	Time to complete the course				Number of steps to complete the course			
	Myo OFF (s)	Myo ON (s)	Abs diff (s)	Perc diff (%)	Myo OFF (-)	Myo ON (-)	Abs diff (-)	Perc diff (%)
P1	166.9	163.1	3.8	2.3	238	232	6	2.5
P2	547.5	480.6	66.9	12.2	391	377	14	3.6
P3	122.0	126.7	-4.7	-3.9	207	202	5	2.4
P5	232.0	223.8	8.2	3.5	245	233	12	4.9
P6	421.8	396.0	25.8	6.1	467	462	5	1.1
P7	339.2	280.1	59.1	17.4	280	236	44	15.7

Table S2. Average heart rate and EMG activity of the vastus lateralis.

ID	Average HR				Average normalized EMG vastus lateralis			
	Myo OFF (bmp)	Myo ON (bmp)	Abs diff (bpm)	Perc diff (%)	Myo OFF (-)	Myo ON (-)	Abs diff (-)	Perc diff (%)
P1	134.0	130.9	3.1	2.3	1.23	1.14	0.09	7.3
P2	140.8	142.4	-1.6	-1.1	1.01	1.02	-0.01	-1.0
P3	152.0	147.5	4.5	3.0	0.98	0.98	0.00	0.0
P5	170.3	178.4	-8.1	-4.8	0.74	0.90	-0.16	-21.6
P6	102.9	106.8	-3.9	-3.8	0.99	1.03	-0.04	-4.0
P7	134.1	127.4	6.7	5.0	1.03	1.13	-0.10	-9.7

Table S3. Average EMG activity of the semitendinosus and the gastrocnemius.

ID	Average normalized EMG semitendinosus				Average normalized EMG gastrocnemius			
	Myo OFF (-)	Myo ON (-)	Abs diff (-)	Perc diff (%)	Myo OFF (-)	Myo ON (-)	Abs diff (-)	Perc diff (%)
P1	0.88	1.11	-0.23	-26.1	0.89	1.00	-0.11	-12.4
P2	0.92	0.75	0.17	18.5	0.95	0.96	-0.01	-1.1
P3					0.92	0.90	0.02	2.2
P5	0.74	0.66	0.08	10.8	0.72	0.83	-0.11	-15.3
P6	1.00	1.05	-0.05	-5.0	0.95	0.96	-0.01	-1.1
P7								

2 Supplementary Figures

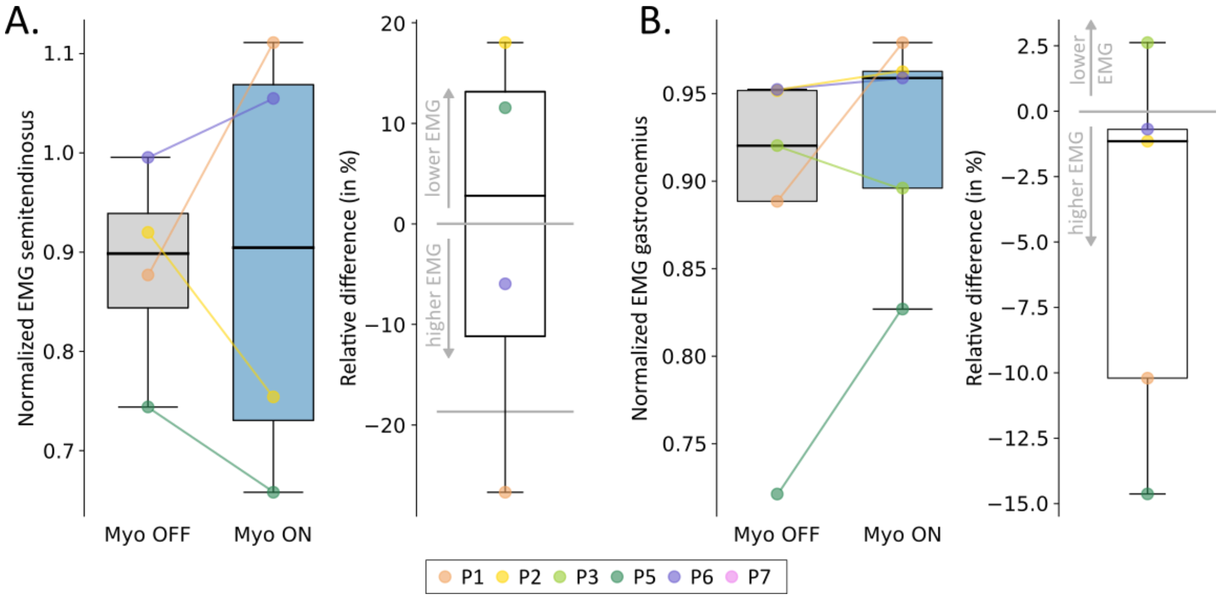

Figure S1. Normalized EMG activity of the semitendinosus and the gastrocnemius. (A) Normalized muscle activity of the semitendinosus over the entire course. (B) Normalized muscle activity of the gastrocnemius over the entire course. Each panel shows the results for the Myo OFF and Myo ON conditions on the left and the percentage differences between the two conditions on the right. Individual responses for each participant are reported using different colors.
